# New Horizons in Plant Photoperiodism

**DOI:** 10.1146/annurev-arplant-070522-055628

**Published:** 2023-02-28

**Authors:** Joshua M. Gendron, Dorothee Staiger

**Affiliations:** 1 Department of Molecular, Cellular and Developmental Biology, Yale University, New Haven, Connecticut, USA; 2 RNA Biology and Molecular Physiology, Faculty of Biology, Bielefeld University, Bielefeld, Germany

**Keywords:** photoperiod, external coincidence, metabolic daylength measurement system, noncanonical photoperiod measurement, photosynthetic photoperiod

## Abstract

Photoperiod-measuring mechanisms allow organisms to anticipate seasonal changes to align reproduction and growth with appropriate times of the year. This review provides historical and modern context to studies of plant photoperiodism. We describe how studies of photoperiodic flowering in plants led to the first theoretical models of photoperiod-measuring mechanisms in any organism. We discuss how more recent molecular genetic studies in *Arabidopsis* and rice have revisited these concepts. We then discuss how photoperiod transcriptomics provides new lessons about photoperiodic gene regulatory networks and the discovery of noncanonical photoperiodmeasuring systems housed in metabolic networks of plants. This leads to an examination of nonflowering developmental processes controlled by photoperiod, including metabolism and growth. Finally, we highlight the importance of understanding photoperiodism in the context of climate change, delving into the rapid latitudinal migration of plant species and the potential role of photoperiod-measuring systems in generating photic barriers during migration.

## 1. INTRODUCTION

The length of daily light phase, the photoperiod, changes in the course of the year due to Earth’s rotation and axial tilt. Plants rely on this predictable variation to optimize their life with seasonal progression. Although phenomena such as seasonal reproduction in animals or seasonal disorders in humans have long been known, progress in research on photoperiod has been largely driven by studies in plants (45, 71, 134). One century ago, Garner & Allard (40) reported on their landmark study in Maryland Mammoth tobacco, which does not flower in summertime unless exposed to extended darkness in the second half of the day. They coined the term photoperiodism for this dependence on the relative duration of day and night.

As our mechanistic understanding of seasonally controlled processes has largely been based on photoperiodic flowering (see Section 1.1), we have far less understanding of photoperiodmeasuring mechanisms controlling nonflowering processes, especially growth. Recently, a boom in transcriptomics has provided insights into the cellular processes that are under the control of photoperiod but has also led to the discovery of noncanonical photoperiod-measuring mechanisms. Importantly, one of these mechanisms, the metabolic daylength measurement system, measures the duration of the day as a function of photosynthetic activity, termed a photosynthetic period, an idea that was proposed nearly forty years ago (19). In addition, plants have mechanisms to measure photoperiod to control hypocotyl elongation and alter the phase of the circadian clock, suggesting that there are a host of photoperiod measurement systems and responses to photoperiod outside of the well-studied photoperiodic flowering system. Because climate change is causing rapid disjunction between daylength and other seasonal signals, such as water availability and temperature, understanding photoperiod measurement systems in plants is paramount to engineering climate-robust crops.

### 1.1. The Road to Photoperiodic Flowering

**Figure 1:**
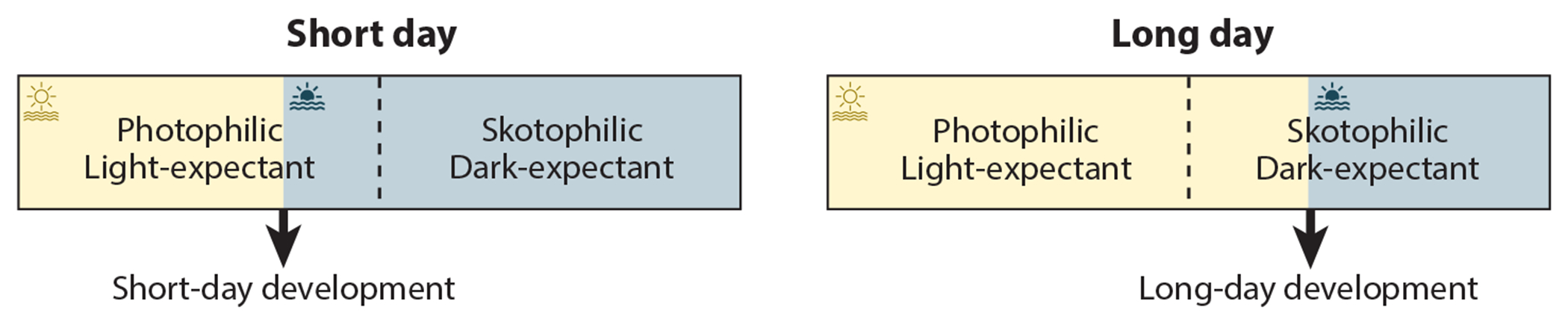
Model for photoperiod measurement. In the course of a 24-h day (*rectangular box*), organisms are sequentially exposed to two states, as indicated by the dashed line. In the first part of the day, the organism is in a photophilic, or light-expectant, state. In the second part of the day, the organism is in a skotophilic, or dark-expectant, state. A 24-h timer, either circadian or hourglass, determines the phasing of the light-expectant and dark-expectant states. Daylight is shown in yellow, and darkness in blue. Daylight extending into the dark-expectant state indicates long-day conditions, inducing flowering in long-day plants and repressing flowering in short-day plants.

Photoperiodic timekeepers measure daylength irrespective of light intensity. In the premolecular era, studies of photoperiodic flowering were foundational for the development of theoretical models describing how an organism could count the hours of light and dark. The German botanist Erwin Bünning (14) was the first to implicate an endogenous rhythm of alternating light-expectant (photophile) and dark-expectant (skotophile) phases in photoperiodic timekeeping (Figure 1). In this model, when daylength is long enough that environmental light surpasses the light-expectant phase and encroaches in the dark-expectant phase, the plant detects that light as a long day (LD). When daylength is short enough that environmental light avoids the dark-expectant phase, the plant detects this as a short day (SD). Once the daylength is measured, the appropriate response is triggered. Bünning’s concept was later refined by Pittendrigh (109) in the external coincidence model of an endogenous rhythm that needs to productively interact with light to trigger the photoperiodic response. Alternatively, in the internal coincidence model, the photoperiodic response results from the interaction of at least two endogenous regulators with their rhythms falling into phase. Light, in this case, has only one function: to synchronize the phases of these rhythms (109).

**Figure 2:**
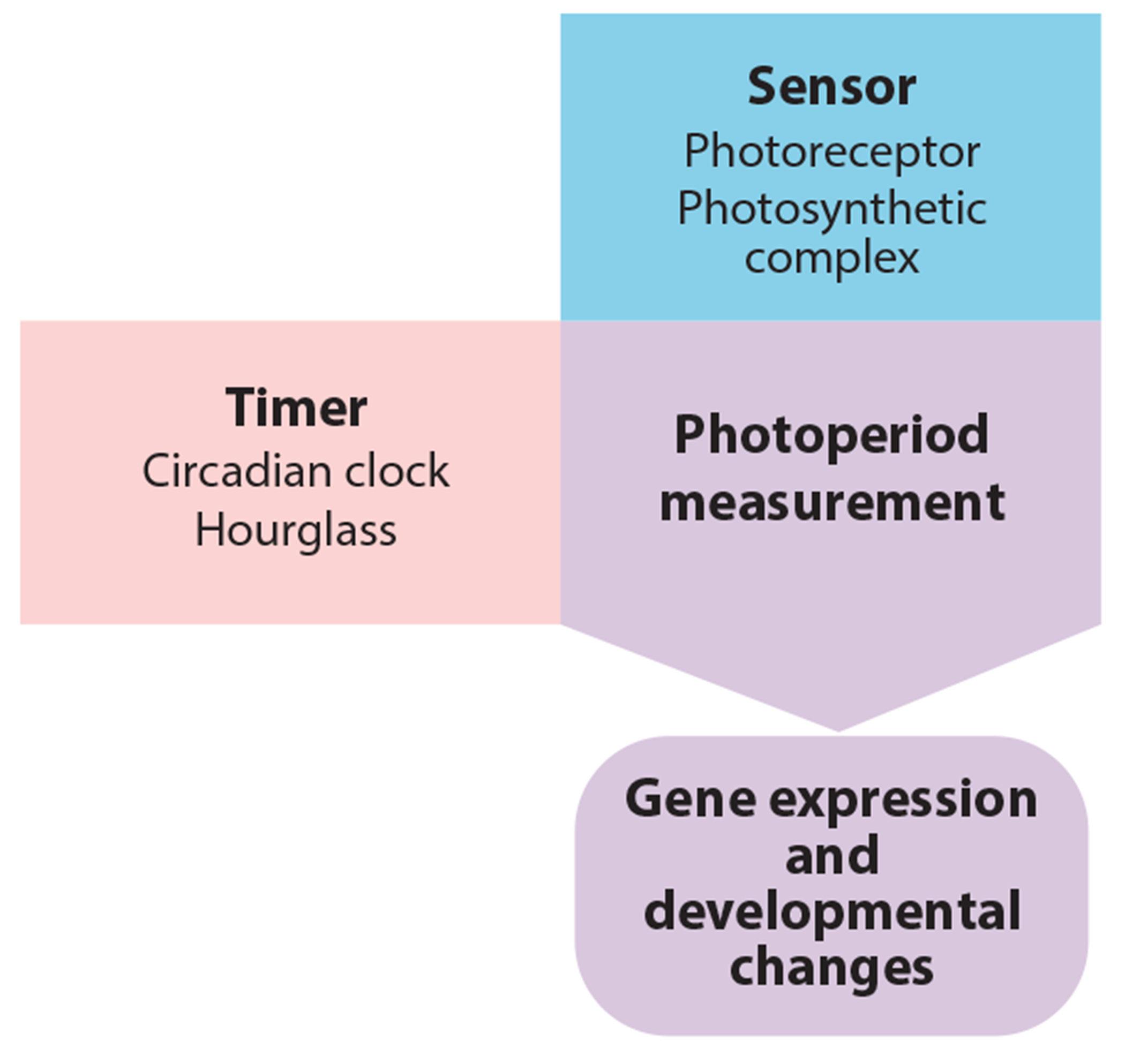
Three components are required for photoperiod measurement: (*a*) a timer (*pink rectangle*), either a circadian clock or hourglass, that sets the light-expectant and dark-expectant phases; (*b*) a sensor (*blue rectangle*) that can distinguish between light and dark (in plants, this can be a photoreceptor or the photosynthetic apparatus); and (*c*) a photoperiod-measuring process (*purple arrow*), whose phasing is set by the timer (establishing light-expectant and dark-expectant states), that functions differently in light and dark. This process then controls physiology and gene expression differentially in short days and long days (*rounded purple rectangle*).

These models rely on an internal biological timer that parses the light-expectant and dark-expectant phases to the appropriate times of the 24-h day. Bünning (14) was convinced that the light- and dark-expectant phases were generated by a genetically encoded oscillatory clock, but hourglass timers based on unidirectional processes were subsequently shown to also act in photoperiodic measurement systems in plants and animals (103, 113) (Figure 2; see also Section 1.3).

### 1.2. The Conceptual Model of the Circadian Clock in *Arabidopsis thaliana*

To date, the presence of an oscillatory clock as postulated by Bunning and Pittendrigh (14, 108, 109) has empirical support in most organisms. This biological clock generates endogenous rhythms with a period of about 24 h, hence designated the circadian clock (from *circa diem*, meaning about one day). The cellular clockwork consists of proteins that are expressed in a temporal series throughout the day and generate their own 24-h rhythm via negative feedback (46). In vascular plants, the blueprint of the clockwork has been resolved in the model plant *Arabidopsis thaliana*. Briefly, the core clock feedback loop involves the morning-expressed transcription factors CIRCADIAN CLOCK ASSOCIATED1 (CCA1) and LATE ELONGATED HYPOCOTYL (LHY) that repress the evening-expressed *TIMING OF CAB EXPRESSION1* (*TOC1*) (2, 122, 146). Additionally, CCA1 and LHY repress *EARLY FLOWERING3* (*ELF3*), *ELF4*, and *LUX ARRHYTHMO* (*LUX*), which form the evening complex (EC), as well as *GIGANTEA* (*GI*). PSEUDORESPONSE REGULATOR9 (PRR9), PRR7, and PRR5 sequentially accumulate throughout the day and repress *CCA1* and *LHY*. The EC indirectly derepresses *CCA1* and *LHY* by downregulation of *PRR7, PRR9,* and *GI*, allowing the cycle to start again the next day (85). In addition to this transcriptional control, the clock components are regulated at the posttranscriptional and post-translational levels to feed environmental information into the clock (52, 128, 153). In other plant species, the clockwork essentially comprises orthologous components with distinct variations (62, 129, 139).

The rhythmically expressed clock proteins in turn cause large parts of the transcriptome to oscillate within a 24-h period and regulate physiological processes, the so-called clock output (see Section 3) (47, 90). In turn, light input to the clock is the prevalent cue synchronizing the circadian clockwork to the day-night cycle in a process known as entrainment (32, 109). Plants rely on multiple photoreceptors to monitor changes in light quality, quantity, and duration. *Arabidopsis* has five phytochromes, among which PhyA detects very-low-fluence red and high-intensity far-red light and PhyB detects red light. Cryptochrome 1 (Cry1) and Cry2 perceive blue light, and both phytochromes and cryptochromes convey light input to the clock (91). Furthermore, the light-oxygen-voltage domain E3-ubiquitin ligase proteins ZEITLUPE and FLAVIN-BINDING KELCH REPEAT F-BOX1 (FKF1) mediate blue light input (126). In addition to setting the photoperiodic response rhythms through clock entrainment, photoreceptors directly report on ambient light to distinguish SDs from LDs (see Section 2.1).

### 1.3. Hourglass Timers in Photoperiod Measurement

Although most plants studied to date use the circadian clock for photoperiodic flowering, there is evidence that some plants use hourglass timers, and keeping this in mind when studying new photoperiod measurement systems is important (Figure 2). In the hourglass model, the length of either day or night is measured by the accumulation of a hypothetical substrate over time, which does not require entrainment, resetting immediately in response to any new dawn or dusk. The process functions for the required amount of time, for instance, 12 h, and then reverts back to the starting point (113). Examples for hourglass timers include the phytochrome photoreceptors. Upon absorption of red light, the inactive Pr (red light–absorbing) form is converted to the active Pfr (far-red light–absorbing) form. In addition to the reversion of Pfr to Pr by absorption of far-red light, active Pfr slowly reverts to inactive Pr in the absence of light, a process known as dark reversion, and serves as an hourglass timer. In SD plants, Pfr would repress flowering, and measurement of the dark phase would begin at dusk, leading to derepression of flowering after a critical duration of darkness when sufficient Pfr has reverted back to Pr (134).

To distinguish between hourglass and circadian clock timers, daylength is kept constant and the dark period is systematically lengthened to provide cycles longer than 24 h in the so-called Nanda-Hamner protocol (97). If the process is controlled by a circadian mechanism, the photoperiodic response would only be triggered in a photoperiod that is a multiple of 24 h [i.e., 12 h light:12 h darkness (12L:12D) or 12L:36D] and not in non-24-h cycles (i.e., 12L:16D or 12L:30D). Such experiments showed that in many, but not all, plant species, photoperiodic flowering is under the control of the circadian clock (134). Importantly, either timer is sufficient to differentially interpret LDs and SDs.

## 2. THE MOLECULAR BASIS OF PHOTOPERIODIC FLOWERING

### 2.1. Long-Day Plants

The molecular details of photoperiodic flower induction are best understood in *Arabidopsis thaliana*, a facultative long-day plant found across the northern hemisphere that flowers steadily earlier when days get longer in spring and summer to finish seed production before the onset of the cold season. The key evidence for the endogenous rhythm of photoperiodic sensitivity proposed by Bunning came from the characterization of *constans* (*co*) mutants that flower late in the LD, i.e., they cannot distinguish inductive long photoperiods from noninductive short photoperiods (111). Molecular analysis implicated the transcription factor CO as the missing link between the circadian clock and photoperiodic timing.

**Figure 3:**
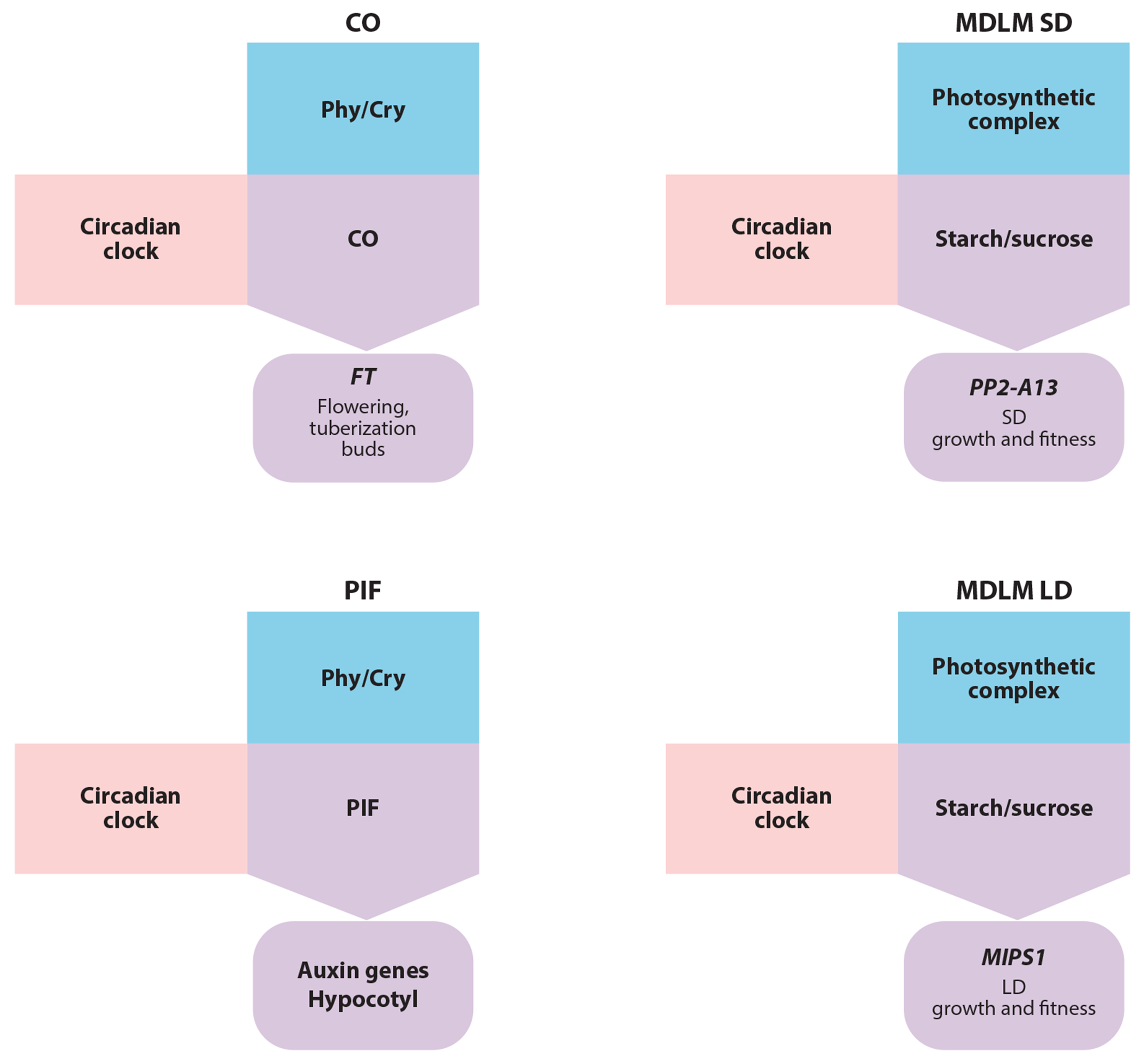
Different types of photoperiod-measuring systems. Each of the photoperiod-measuring systems has a timer and a light sensor involved. For the CO/FT regulon, the timer is the circadian clock and the light sensors are phytochromes, cryptochromes, and FKF1. *CO* mRNA is regulated by the circadian clock and CO protein stability is regulated by light. CO activates *FT* expression, which controls flowering, tuberization, and bud set. For the PIF regulon, the timer is the circadian clock and the light sensors are phytochromes. The circadian clock controls *PIF* mRNA, and light destabilizes the PIF protein. PIFs activate auxins and other growth-promoting genes to increase hypocotyl length in short days. The MDLM system uses the circadian clock as the timer but uses photosynthesis as the light sensor. The circadian clock and photosynthesis combine to control the accumulation and degradation of starch, which results in differential sucrose levels in the dark in SDs and LDs. The MDLM system supports growth and fitness through inducing expression of *PP2-A13* in SDs and *MIPS1* in LDs. Abbreviations: CO, CONSTANS; Cry, cryptochrome; *FT*, *FLOWERING LOCUS* *T*; MDLM, metabolic daylength measurement; *MIPS1, MYO-INOSITOL-1-PHOSPHATE SYNTHASE 1*; mRNA, messenger RNA; LD, long day; Phy, phytochrome; PIF, phytochrome interacting factor; *PP2-A13, PHLOEM PROTEIN 2-A13*; SD, short day.

Exquisite regulation of CO at the transcriptional and posttranslational levels mediates induction of the floral activator FLOWERING LOCUS T (FT) (130, 155) (Figure 3). The *CO* messenger RNA (mRNA) undergoes circadian oscillations with a waveform reflecting the light-expectant and dark-expectant phases of a photoperiodic measurement system. CO protein that is translated in the dark is subject to proteasomal degradation, mediated by a ubiquitin ligase complex consisting of CONSTITUTIVE PHOTOMORPHOGENIC1 (COP1) and SUPPRESSOR OF PHYTOCHROMEA (SPA1) (70, 141). Thus, CO protein serves as an external coincidence sensor activating *FT* expression in inductive LDs (4, 118).

A second mechanism relies on the phasing and activity of FKF1 and GI to properly shape the *CO* mRNA expression profile. *GI* oscillations peak earlier in SDs than FKF1 oscillations, whereas in LDs they are coexpressed at the end of the light phase. FKF1 is activated by blue light at the end of the day in LDs, promoting interaction with GI and causing degradation of the *CO*-repressing CYCLING DOF FACTORs (CDFs), thus allowing *CO* transcription (56, 121). Collectively, this mechanism shows features of both internal and external coincidence: Synchronization of the endogenous *GI* and *FKF1* rhythms in LDs allows degradation of CDFs to create the *CO* transcript pattern, and regulation of FKF1 activity by blue light and of COP1/SPA1 activity through CRYs at the peak of *CO* oscillations allows CO accumulation, ultimately culminating in *FT* expression at the end of the day.

It has long been known that the photoperiod is perceived in leaves and communicated by a mobile signal that travels to the shoot apical meristem (SAM). While the concept of florigen assumed the existence of hormone-like substances (17), *Arabidopsis* molecular genetics unearthed *FT*, which encodes a mobile protein made in phloem companion cells and is transported to the SAM to act as the florigen (25, 61, 83). There, it interacts with the FD transcription factor to activate the expression of *SUPPRESSOR OF OVEREXPRESSION OF CONSTANS1* (*SOC1*) and *FRUITFUL* to initiate SAM reprogramming (1, 149) (see Section 3.1).

Among the LD plants are also temperate grasses, including the important crops *Hordeum vulgare* (barley) and *Triticum aestivum* (wheat) (62, 139). Flowering in LDs is similarly mediated via the upregulation of *FT*-like genes (15), albeit distinct features in the signaling mechanisms are found between *Arabidopsis* and grasses. PhyA and PhyB have a major influence on CO stability in *Arabidopsis*, whereas PhyC plays an important role in the photoperiodic response in barley and wheat (22, 100, 106, 141). In *Brachypodium distachyon*, a model for grass functional genetics, PhyC conveys light information to the EC component ELF3 for photoperiodic flowering (39). Growing *Brachypodium* in non-24-h light-dark cycles suggested that the photoperiodic response is determined by the absolute duration of the dark phase, as predicted for an hourglass timing mechanism (113). Gao et al. (39) therefore revisited the concept of dark reversion of active Pfr to inactive Pr in the absence of light as an hourglass timer (see Section 1.1). 72% of Pfr remained at the end of the night in LDs as opposed to 37% in SDs, suggesting that the level of active Pfr in the night indeed may determine activation of the photoperiodic regulator ELF3 (39, 142).

### 2.2. Short-Day Plants

Plants growing at lower latitudes are often SD plants, flowering when the photoperiod falls below a critical daylength. Thus, they tend to flower in the shorter days of autumn to escape high temperatures in summer. A common theme between LD and SD plants is the transcriptional upregulation of florigens in inductive photoperiods, whereas distinct molecular features provide the basis for the differential daylength responses. Furthermore, many SD plants set a critical daylength for floral transition in contrast to the gradual accumulation of florigen in LD plants (132, 134). This was corroborated by simulations revealing that *FT* does not display a critical daylength threshold in *Arabidopsis* (118).

In rice, *Heading date 3a* (*Hd3a*), an *FT* ortholog, promotes heading in inductive SDs, and *Rice FT-like 1* (*RFT1*) is necessary for heading in noninductive LDs (69). *Heading date 1* (*Hd1*), the *CO* ortholog, is predominantly expressed in the night in SDs and activates *Hd3a* expression to promote heading (49). In noninductive LDs, *Hd1* is expressed from night until dawn, and Hd1 accumulating in the light is converted to an inhibitor of *Hd3a* transcription by red light–activated PhyB signaling, acting as floral repressor (59). Thus, *Hd1* follows a circadian rhythm, similar to *Arabidopsis CO*, but has opposing effects on the *FT* ortholog *Hd3a* (154). Notably, *Arabidopsis* CO was recently shown to repress flowering in SDs apart from its flowering-promoting function in LDs, suggesting that, like rice Hd1, it is bifunctional (78).

In addition to the conserved *Hd1/Hd3a* regulon, a monocot-specific pathway comprising *Grain number and heading date7* (*Ghd7*) and *Early heading date1* (*Ehd1*) regulates heading. Ehd1 activates *RFT1* and *Hd3a* both in SDs and LDs (29). *Ehd1* in turn is repressed by Ghd7, which increases upon lengthening of the photoperiod, thus delaying heading in LDs (104, 152). This monocot-specific cascade is controlled by two distinct photosensitive phases regulated by the circadian clock (58). When the photoperiod exceeds the critical daylength, red light signaling induces *Ghd7*, which in turn counteracts the blue light–dependent *Ehd1* induction, delaying heading. When the photoperiod is shorter than the critical daylength, the sensitive phase for red light–mediated induction of the *Ghd7* repressor is shorter, allowing acute *Ehd1* expression triggered by morning blue light for *Hd3a* induction and heading (29, 58, 67, 104). This toggle switch–like on/off transcriptional reaction of the florigen genes *Hd3a* and *RFT1* contributes to flowering in response to the critical daylength and allows rice to recognize subtle changes in daylength near the equator.

In the SD plant *Ipomoea nil* (formerly *Pharbitis nil*, Japanese morning glory), flowering is controlled by the length of the night, and night breaks of 5 min of red light illumination impair flowering (117). The sensitivity of these plants to the night breaks shows features of external coincidence, and this photoperiodic response rhythm appears to be set at dusk (79). At the molecular level, *PnFT1* and *PnFT2*, the orthologs of *FT*, are expressed specifically in SDs with a rhythm set at dusk so that *PnFT* is phased to a constant time after dusk (48). Accordingly, accessions with differences in the critical night length responses express *PnFT* at different times after dusk (48). This is in contrast to the known circadian rhythms in *Arabidopsis* that are regulated by an oscillator entrained by dawn and dusk but resembles *Arabidopsis elf3* mutants that entrain rhythms at dusk and become arrhythmic in constant light (87). Interestingly, *PnFT* expression is not directly correlated with *PnCO* expression. Thus, daylength measurement in *Ipomoea* utilizes a mechanism that is different from that of *Arabidopsis* or rice.

## 3. PHOTOPERIODIC TRANSCRIPTOMES

### 3.1. Gene Expression Changes During Photoperiodic Flowering in *Arabidopsis*

The transition to flowering marks a grand rearrangement of the developmental and metabolic programs of the plant that is conducted in order to support offspring fitness. In the last few decades, the gene regulatory networks underlying these massive rearrangements have been revealed through transcriptomic experiments.

**Figure 4:**
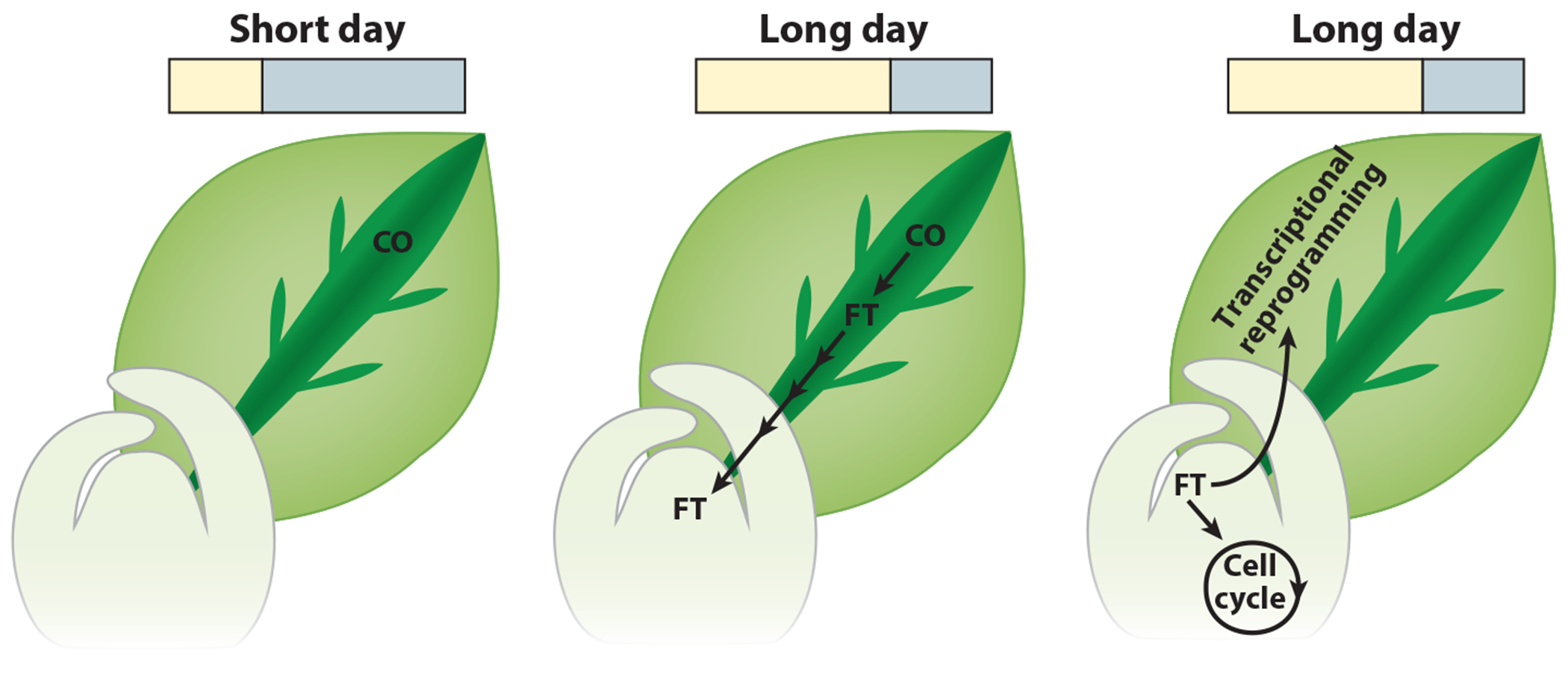
Schematic of transcriptional cascade triggered by the CO/FT regulon. In *Arabidopsis* in short days, CO protein is inactivated, preventing the activation of *FT* expression. Upon transfer to long days, CO is stabilized in the transport tissue and activates *FT* transcription, and FT translocates to the meristem. FT promotes the transition to flowering, which is accompanied by a burst of expression in cell cycle genes. Additionally, transcriptional reprogramming of the rest of the plant occurs to support the transition from vegetative to reproductive development. Abbreviations: CO, CONSTANS; FT, FLOWERING LOCUS T.

*Sinapis alba* (white mustard) was one of the first models used to study photoperiodic control at the molecular level, as exposing SD-grown plants to a single LD elicited a highly synchronized floral transition of the SAM, making it ideal to explore early gene expression changes. Subtractive complementary DNA hybridization upon lengthening of the photoperiod identified early events in the SAM (88) and the leaves upon lengthening of the photoperiod (50, 51) and identified an ortholog of *SOC1* as an early response gene. After the discovery of CO at the core of the photoperiod-sensing pathway in *Arabidopsis*, subtractive hybridization of a transgenic line with steroid-inducible *CO* expression showed that *FT* and *SOC1* are both rapid response genes required for photoperiodic flowering control by CO (119). Subsequently, microarray analysis of the leaves of a *co* mutant confirmed that a major role for CO is to control *FT* expression in leaves (149) (Figure 4). Together, these data suggested that CO has few targets but those targets are at the core of the vegetative-to-floral transition.

Subsequent to the activation of *FT* by CO, FT travels from the leaves to the meristem, and the shift from vegetative to developmental growth is followed by a “burst in expression of cell division,” marking the transition to a rapidly expanding floral meristem (66). Using a transgenic line that constitutively expresses *FT* in the meristem, researchers showed that reciprocal communication occurs from the reproductive meristem back to the vegetative tissues, as thousands of genes are misregulated in the leaves even when FT is restricted to the meristem (30). Coming full circle, RNA sequencing (RNA-seq) of whole *co* mutant plants demonstrated that many genes are misexpressed throughout the plant, suggesting that preventing the transition to flowering disrupts an extensive rearrangement of gene regulatory networks (43).

To obtain a tissue-level spatial resolution of the photoperiod-responsive transcriptome, Tian and coworkers (136) analyzed epidermal cells and vascular companion cells separately via isolation of nuclei tagged in specific cell types (INTACT). Cross-referencing gene expression with chromatin accessibility monitored with transposase-accessible chromatin using sequencing (ATAC-seq) data revealed that LDs result in general increases in chromatin accessibility, while RNA-seq showed differential gene expression changes in the vasculature and mesophyll cells. For instance, circadian clock and signaling genes change more dramatically in the vasculature, while energy-related genes change in the mesophyll, providing evidence for cell- and tissue-specific photoperiodic gene networks. This was in concordance with tissue-specific ATAC-seq that showed vascular-specific, mesophyll-specific, and overlapping LD-induced accessible chromatin regions, with vasculature having roughly twice the number of photoperiod-specific accessible chromatin regions.

In summary, transcriptome studies have revealed a transcriptional cascade starting with CO inducing *FT* in the vasculature, followed by the meristem-specific induction of growth-promoting genes, and ending with whole-plant transcriptional reprogramming in support of the transition to reproduction and then production of offspring.

### 3.2. General Photoperiodic Gene Expression Changes in *Arabidopsis*

At least one-third of the *Arabidopsis* transcriptome is under the control of the circadian clock or displays diurnal rhythms, and recently researchers estimated that one-third of the transcriptome also changes in response to photoperiod (26, 47, 74, 90, 92). The first glimpses of the full scope of photoperiod-regulated genes and cellular processes came from a microarray time-course analysis of SD- and LD-grown *Arabidopsis* (90, 92). These early forays demonstrated that photoperiod-dependent changes can occur as changes in amplitude (as observed for *FT*) or phase (as observed for clock genes) and are best captured through collecting data over at least 24 h (Figure 4). Microarray was again employed for the analysis of gene expression changes in *Arabidopsis* in response to a wider range of photoperiods (4-, 6-, 8-, 12-, and 18-h photoperiods), although daily sampling density was restricted to dusk and dawn (37). This study confirmed the phase delay of core clock transcripts, such as *CCA1* and *LHY*, in lengthening photoperiods but also identified key photoperiod-regulated genes such as *PRODUCTION OF ANTHOCYANIN PIGMENT 1* and *SNF1-RELATED PROTEIN KINASE REGULATORY SUBUNIT BETA-1* and cellular processes such as primary and specialized metabolism, energy sensing, and protein translation.

### 3.3. Photoperiodic Gene Expression Beyond *Arabidopsis thaliana*

High-throughput sequencing has also facilitated the study of photoperiod-controlled gene expression in plant systems outside of *Arabidopsis thaliana*. Seasonal gene expression changes and methylation state were assessed in *Arabidopsis halleri*, a perennial relative of *Arabidopsis thaliana* that was grown in natural environments and thus exposed to a wide array of seasonal variables apart from photoperiod (57, 96). Importantly, samples were collected once a week for 2 years but included the collection of 2-h resolution time-course samples from the equinoxes and the solstices. This study showed that photoperiod controls the phase of expression of core clock genes, even in the wild, but also showed that nearly 1,800 genes have amplitude or phase changes controlled by the seasons. In addition, bisulfite sequencing hinted that methylation of specific genomic loci is seasonally controlled, although the bulk of methylation is stable throughout the year. This highlighted the importance of photoperiod for gene expression and epigenetic changes in an environment where the plant is challenged by the full complement of biotic and abiotic changes accompanying seasons.

Photoperiod gene expression studies in wheat, *Medicago, Panicum hallii*, sugarcane, and soybean showed that, similar to previous studies, metabolic, clock, and flowering-time genes are regulated seasonally, confirming the idea that there are core cellular networks that respond to photoperiod in a wide array of plant species (80, 120, 135, 148).

### 3.4. Data Mining Transcriptomes for Mechanisms of Photoperiod-Regulated Gene Expression

Available photoperiodic gene expression data sets and the development of new tools to analyze this data have resulted in the successful identification of new photoperiod-measuring mechanisms. A meta-analysis using *Arabidopsis* photoperiod data revealed that PhyA is important for photoperiod sensing at dawn, especially in SDs (123). Furthermore, photoperiod gene expression studies in wheat noted that PhyB and PhyC do not control the expression of a large number of photoperiod-regulated genes, while studies in *Brachypodium* helped reveal a role for PhyC in photoperiod measurement (39, 65, 107). This highlights the idea that photoperiodic gene expression changes are likely occurring through multiple mechanisms and can recruit different phytochromes for photoperiod sensing or possibly work independently of the phytochromes.

The aforementioned *Arabidopsis* photoperiod time-course RNA-seq data set was also used to suggest that a variety of photoperiod transcriptional networks are likely operating in *Arabidopsis* (74). The data set was generated with *Arabidopsis* grown in three photoperiods (16L:8D, 12L:12D, and 8L:16D) with 4-h resolution across 24 h of each photoperiod. Using an rDEI calculation (rDEI = sum of 24 h of expression in condition one/sum of 24 h of expression in condition two), pattern clustering, and *cis*-element analyses, researchers identified a multitude of photoperiod-responsive coexpression clusters. These included known clusters such as clock-regulated genes that phase shift but also unknown clusters such as a group of genes encoding ribosomal proteins that respond to photoperiod with a specific expression trough. This work suggests that many photoperiod measurement systems are likely working in parallel to control the expression of genes involved in specific biological and cellular processes (74).

**Figure 5:**
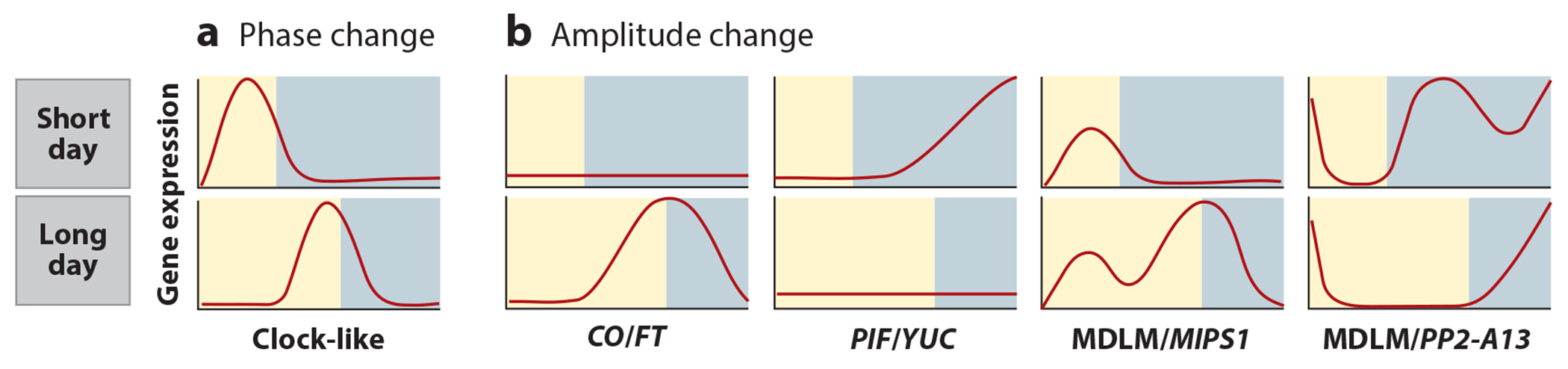
Schematic of photoperiodic gene expression patterns. Photoperiodic changes in gene expression can occur as changes in phase or amplitude. (*a*) When days get longer, the phase of core clock genes and clock-controlled genes delay. (*b*) Amplitude changes have been shown to occur in four ways. In the *CO/FT* regulon in long-day plants, *FT* expression is nearly undetectable in short days, but upon transfer to long days a dusk-phased pattern of *FT* expression appears. PIF target genes increase in amplitude at the end of a long night to promote hypocotyl elongation. Genes controlled by an MDLM system have a similar amplitude change as *FT* or PIF targets but have a second peak in expression that is present in any photoperiod. Thus, genes such as *PP2-A13* and *MIPS1* go from monophasic to biphasic, depending on the photoperiod. Daylight is shown in yellow, and darkness in blue. Abbreviations: *CO*, *CONSTANS*; *FT*, *FLOWERING LOCUS* *T*; MDLM, metabolic daylength measurement; *MIPS1, MYO-INOSITOL-1-PHOSPHATE SYNTHASE* *1*; PIF, phytochrome-interacting factor; *PP2-A13, PHLOEM PROTEIN 2-A13*.

**Figure 6:**
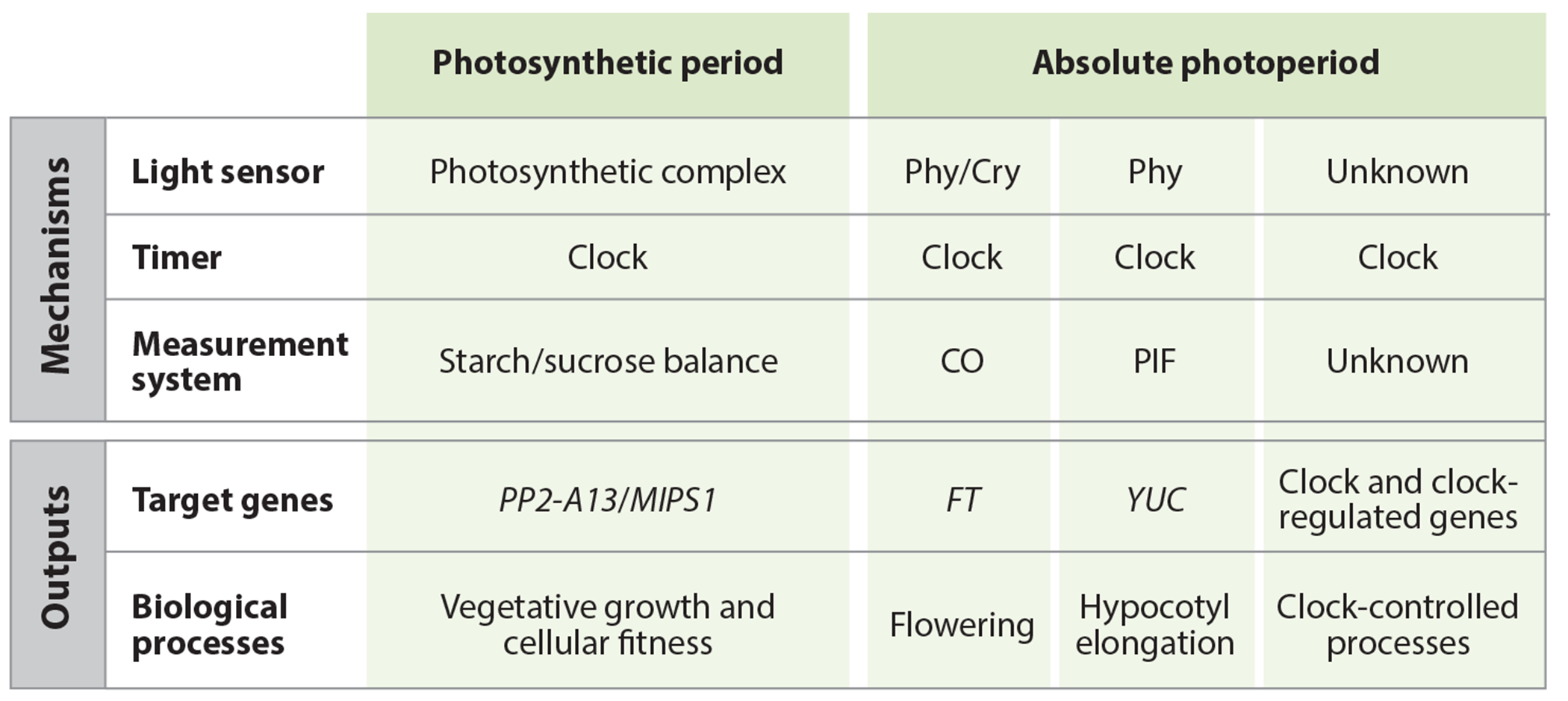
Summary of photoperiod measurement mechanisms in plants. The two types of photoperiods that plants are known to measure, photosynthetic period and absolute photoperiod, are shown at the top with the mechanisms that measure each photoperiod type below. For each mechanism, the light sensor, timer type, and photoperiod-measuring system are listed. In addition, known target genes and biological processes are listed (below). Abbreviations: CO, CONSTANS; Cry, cryptochrome; *FT, FLOWERING LOCUS T; MIPS1, MYO-INOSITOL-1-PHOSPHATE SYNTHASE 1*; Phy, phytochrome; PIF, phytochrome-interacting factor; *PP2-A13, PHLOEM PROTEIN 2-A13; YUC*, *YUCCA*.

In support of this idea, the original photoperiod time-course microarray data (90) were reanalyzed using the rDEI calculation that allowed for time-independent identification of photoperiod-induced genes. Clustering of these genes based on their daily patterns of expression in different photoperiods revealed a biphasic expression pattern with high expression in SDs, a pattern first recognized in the original analyses of microarray data (76, 90) (Figure 5; see also Figure 6). Deeper examination of this biphasic expression pattern led to the discovery of a noncanonical photoperiod-measuring system housed in the metabolic networks of plants (see Section 4).

In sum, transcriptomics is particularly important for understanding daily and seasonal timing mechanisms in plants. Perhaps the efficacy of these approaches lies in the nature of daily timing mechanisms, which are driven by closed 24-h loops, allowing for a full survey of the scope of gene expression changes. Thus, best practices for generating new photoperiod transcriptomic data should include at least 24 h of time points with the highest temporal resolution possible.

## 4. NONCANONICAL PHOTOPERIOD-MEASURING SYSTEMS

Studies of flowering time in plants are responsible for our current understanding of the fundamental principles of photoperiod measuring in all organisms (see Section 1.1), but the canonical CO/FT regulon for flowering cannot account for the totality of photoperiod-dependent changes. A few lines of evidence suggest this. Firstly, early expression studies suggested that CO is regulating few genes in the leaves, but many genes change their expression in different photoperiods (see Section 3). Secondly, photoperiodic flowering is dispensable, with some plant species being day neutral for flowering. Thirdly, SD plants flower faster in SDs but often grow faster in LDs, suggesting that these two important processes can be disconnected in time. Fourthly, on one hand, mutations in CO lead to defects in photoperiodic flowering but seem to have little effect on other processes, while on the other hand circadian clock mutants have defects in flowering, growth, metabolism, and a multitude of photoperiod-controlled cellular processes, suggesting that there may be nonflowering photoperiodic processes that require proper clock function (46, 122, 146). Fifthly, the photoperiodic flowering networks are often localized in transport tissues of the plant, but photoperiodic changes occur in nontransport tissues, although reciprocal regulation was discussed above. This is exemplified by the tissue-specific RNA-seq experiment showing differential regulation of genes in the mesophyll and transport cells and suggesting different photoperiod timekeeping mechanisms based on cell type (136). In the following section, we describe the current understanding of noncanonical photoperiod-measuring systems with updated information on the known molecular components for each.

### 4.1. The Metabolic Daylength Measurement System

In *Arabidopsis*, CO causes the induction of genes in LDs, such as *FT*. Mounting evidence suggests that photoperiodic timekeeping is not constrained to targets of CO and that there are photoperiod measurement systems that can induce gene expression in LDs or SDs independently of CO. Recently, the metabolic daylength measurement (MDLM) system was shown to control the expression of genes in both SD and LD conditions (41, 76, 145). Initially, photoperiod microarray data were used to show that a class of genes is expressed in a biphasic manner preferentially in SDs (76) (Figure 5). One of these genes, *PHLOEM PROTEIN 2-A13* (*PP2-A13*) was then shown to support SD-specific growth and fitness. A *PP2-A13*_*promoter*_*:LUCIFERASE* reporter was then used to demonstrate that *PP2-A13* expression is under the control of a CO-independent photoperiod-measuring system and that this system is housed in the plant metabolic networks, hence the name metabolic daylength measurement (MDLM) system. In this system, light is sensed by the photosynthetic apparatus, which then acts to produce sucrose and starch. The circadian clock functions in this system to relay time-of-day information to accurately control the accumulation and degradation of starch (36, 93, 94). In SDs, the plant saves most of the photosynthetic product as starch, resulting in low nighttime sucrose levels, whereas in LDs, more of the starch is converted to sucrose, resulting in high nighttime sucrose levels. The sucrose is converted into a transcriptional signal through an unidentified sugar-signaling network, which results in the induction of genes, such as *PP2-A13*, in the low-sucrose nights of SDs to support vegetative growth and fitness (Figure 4). To demonstrate the importance of starch/sucrose partitioning, mutants in starch production or starch breakdown lack the ability to measure seasons accurately and properly control MDLM-regulated genes, such as *PP2-A13*.

This work was then expanded to show that the MDLM system is bifunctional and can also control induction of genes in LDs (145) (Figure 4; see also Section 5.3). In this study, the MDLM system promotes LD expression of *MYO-INOSITOL-1-PHOSPHATE SYNTHASE 1* (*MIPS1*), which is required to support leaf growth exclusively in LDs (145). It does this by increasing free sucrose levels in the light during the latter part of an LD. The *mips1* mutant is unable to generate fresh weight in LDs but amazingly does not affect leaf organogenesis or flowering time, suggesting that the MDLM and CO photoperiod-measuring systems are working in parallel.

**Figure 7:**
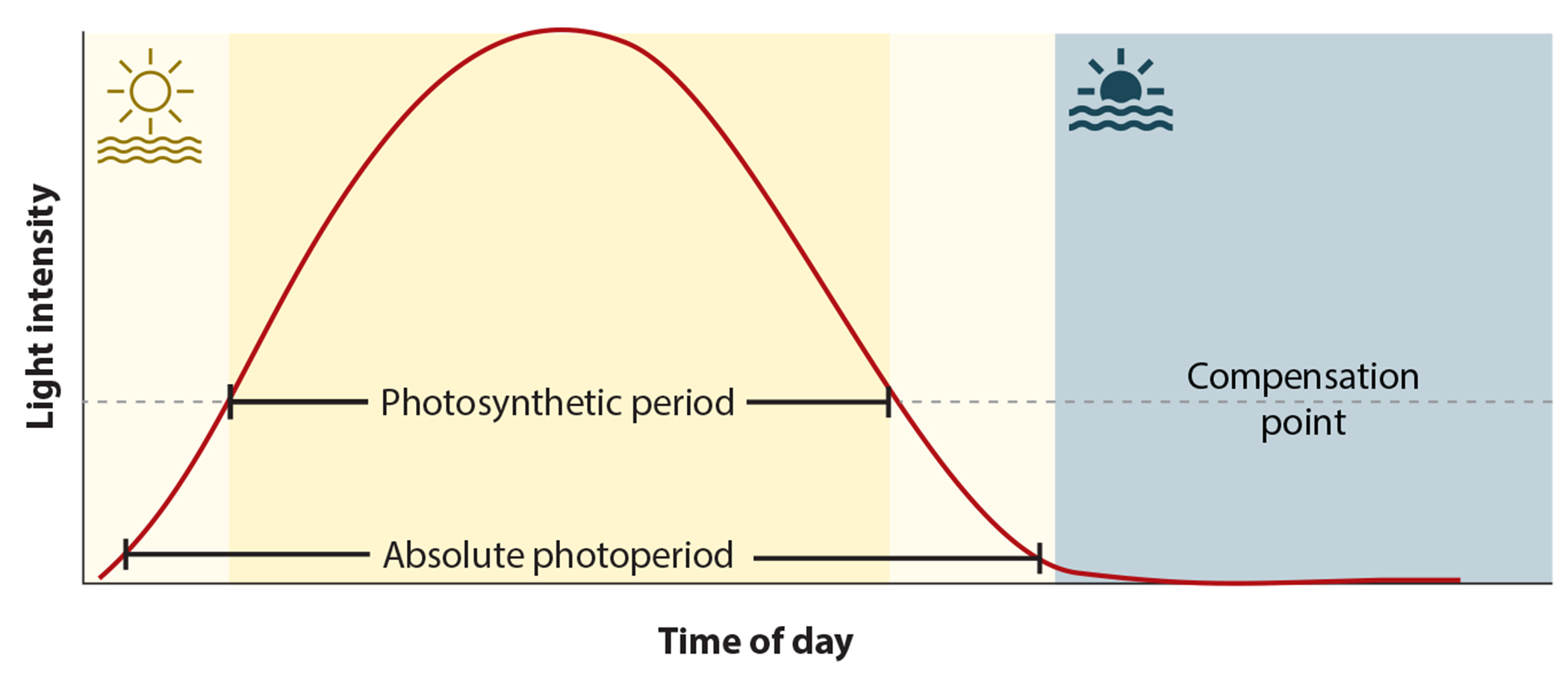
Schematic of photoperiod types measured by plants: the absolute photoperiod and the photosynthetic period. The absolute photoperiod is the time of day when the photoreceptors are active. This can occur at very low light levels and is determined by the light sensitivity of the photoreceptors. The CO/FT and PIF photoperiod measurement regulons are controlled by this type of daylength; thus, hypocotyl growth, flowering, tuberization, and bud set are responsive. The photosynthetic period is the time of the day when light is above the compensation point and the plant is able to fix carbon. The photosynthetic period is sensed by the photosynthetic complex and controls the MDLM system. In turn, genes such as *PP2-A13* and *MIPS1* are regulated by the photosynthetic period and are responsible for supporting vegetative leaf growth, fitness, and yield. Daylight is shown in yellow, and darkness in blue. Abbreviations: CO, CONSTANS; FT, FLOWERING LOCUS T; MDLM, metabolic daylength measurement; *MIPS1, MYO-INOSITOL-1-PHOSPHATE SYNTHASE 1*; PIF, phytochrome-interacting factor; *PP2-A13, PHLOEM PROTEIN 2-A13*.

**Figure 8:**
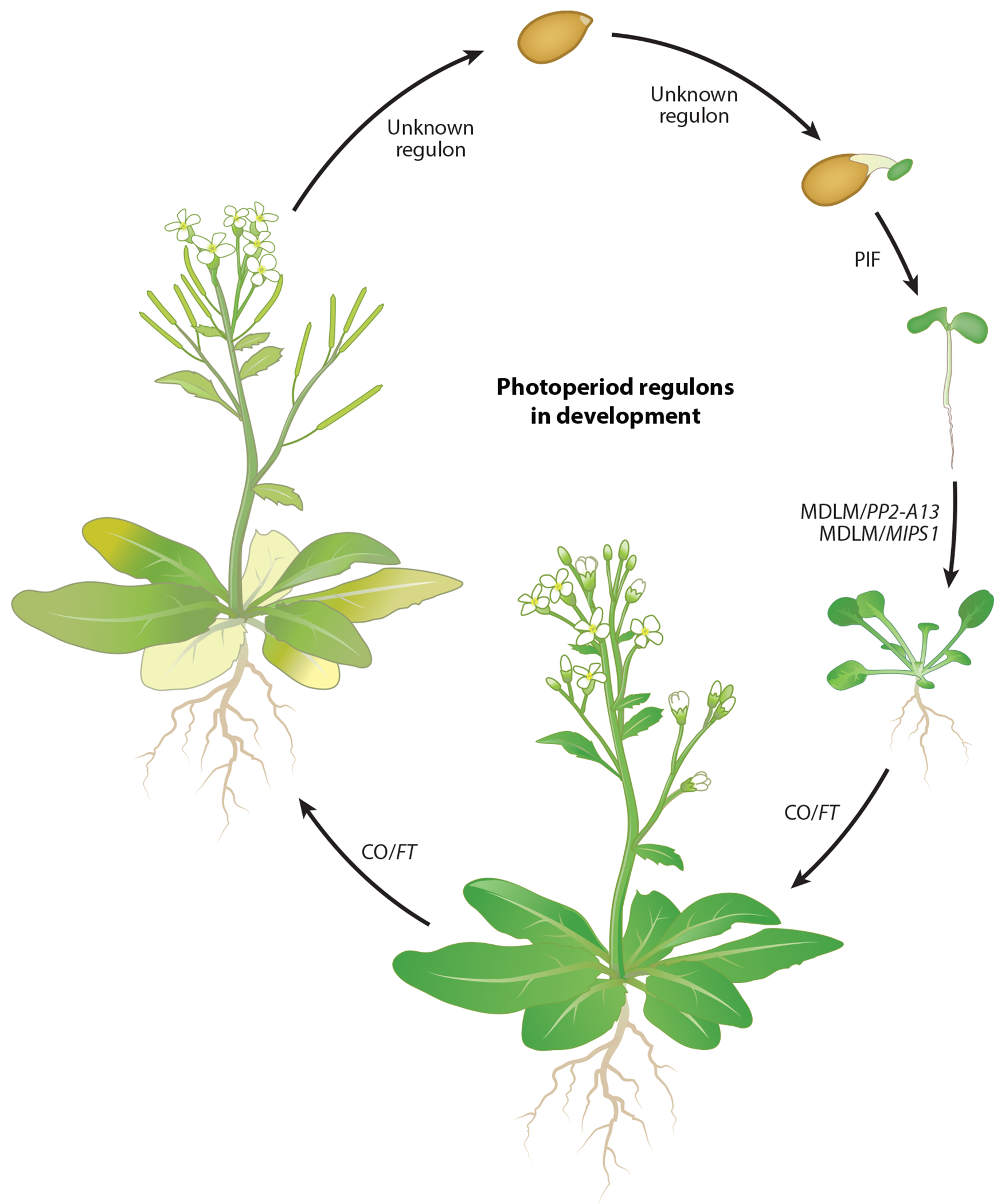
Photoperiod regulons throughout plant development. The photoperiod measurement systems that control germination in the seed or through maternal effect are not known. Subsequent to germination, the PIF regulon controls photomorphogenic hypocotyl growth to ensure that the plant can reach the light for photosynthesis. After reaching light, the plant transitions to growing leaves, and the MDLM system supports optimal vegetative leaf growth in either short or long days. The transition to reproduction, and eventually senescence, is controlled by the CO/*FT* regulon. Abbreviations: CO, CONSTANS; *FT, FLOWERING LOCUS T*; MDLM, metabolic daylength measurement; *MIPS1, MYO-INOSITOL-1-PHOSPHATE SYNTHASE* *1*; PIF, phytochrome-interacting factor; *PP2-A13, PHLOEM PROTEIN 2-A13*.

Perhaps most importantly, the discovery of the MDLM system shows that plants can measure two types of photoperiod, the absolute photoperiod and the photosynthetic period, a concept suggested nearly forty years ago (19) (Figure 7). The MDLM system is sensing the photosynthetic period, that is, it measures the number of hours per day in which light is above the compensation point, allowing the plant to fix carbon (19, 33, 89). This was demonstrated in an experiment akin to a classic night break experiment, which showed that light above the compensation point during the middle part of the day could promote growth and induce the *mips1* mutant growth defect, but light above the compensation point at the beginning of the day could not. Conversely, the CO/FT photoperiodic timekeeper relies on phytochromes for light sensing, which detect light at very low intensities, suggesting that the absolute photoperiod is measured (Figure 7). This ability to detect absolute photoperiod and photosynthetic period makes sense. It allows plants to ensure reproduction in a particular season every year but also to adjust their own physiological growth status in a diversity of light environments (Figure 8).

### 4.2. The PIF Coincidence Mechanism

In addition to the MDLM system, there is another CO-independent mechanism controlling SD induction of genes that was discovered by studying rhythmic hypocotyl growth. *Arabidopsis* hypocotyls elongate in a photoperiodic manner, growing faster in SDs than in LDs. By using high temporal resolution hypocotyl growth assays, researchers have shown that hypocotyl elongation is phased to near dawn by the circadian clock (102). The circadian clock was then shown to control the expression of the red light-signaling and growth-promoting transcription factor genes, *PHYTOCHROME-INTERACTING FACTOR 4* (*PIF4*) and *PIF5*, phasing their expression to the end of the night (Figure 3). In turn, PIF4 regulates the expression of transcription factors and auxin synthesis genes that control hypocotyl elongation (81, 82, 101). Coincidence occurs when light triggers the ubiquitination and degradation of the PIF proteins by the BLADE ON PETIOLE 1 and BLADE ON PETIOLE 2 BTB-ankyrin E3 ubiquitin ligases. This keeps their levels low in LDs, but allows for accumulation in SDs, precipitating hypocotyl elongation (102, 157).

### 4.3. Phase Shifting of the Circadian Clock–Controlled Genes and Other Transcriptional Responses to Photoperiod

In addition to the MDLM and PIF photoperiod-measuring systems, we know that circadian clock–controlled genes exhibit phase shifts in response to changing photoperiods, but little is known about the molecular mechanisms that drive these shifts. Furthermore, transcriptomics suggests that there are additional photoperiod-measuring systems controlling gene expression (74). Undoubtedly, future efforts will focus on understanding the full breadth of photoperiod-measuring systems in plants, revealing how plants coordinate a range of developmental processes in response to seasonal changes in daylength.

## 5. PHOTOPERIODIC CONTROL OF DEVELOPMENT

Plants undergo a series of developmental transitions, starting with the germination of the seed and progressing to the generation and expansion of leaves for primary metabolism, reproduction, and then senescence (Figure 8). New and classic evidence suggests photoperiod may play a role in controlling many of these important developmental processes, and we are beginning to link known photoperiod measurement systems to the various developmental responses. The following sections cover some major developmental responses to photoperiod and link these to known photoperiod measurement systems when possible.

### 5.1. Germination

Seasonal timing of germination has a clear impact on seedling fitness, but little is known about the molecular mechanisms that control germination in response to photoperiod. This is likely due to the relatively small effects of photoperiod on seedling germination in the genetic model, *Arabidopsis thaliana*. In *Arabidopsis*, the photoperiod that seeds experience at germination has less influence than the photoperiod experienced by the mother plant (55). Plants grown in SDs have higher offspring germination rates than counterparts grown in LDs. Maternal effects do have control over germination rates in other plant species, but the photoperiod experienced by the seeds also has larger impacts on germination in species such as birch (8; reviewed in 68). This effect of the maternal and seed-experienced photoperiods, in combination with vernalization, the exposure to winter temperatures, can serve to optimally time germination in plants with different lifestyles (e.g., winter annuals, spring/summer annuals). In a natural environment, genetic variation in response to maternal photoperiod may allow optimal timing of germination in habitats with different climates, and molecular genetics should be employed to understand the underlying networks that control photoperiodic seed germination. Currently, little is known about what potential photoperiod-measuring system may be operating in seeds to control germination.

### 5.2. Metabolism

As photoautotrophic organisms, postgermination plants become metabolically dependent on ambient light for most developmental processes. Alignment of the circadian clock with the diurnal environment is required for *Arabidopsis* fitness, in part through coordination of photosynthetic energy capture (28). Shortening photoperiods represent a potential energy-limiting environment for plants, while lengthening photoperiods signal times when energy is replete. In response, photosynthetic capacity changes with the photoperiod through alterations in leaf size and shape, chlorophyll content, stomatal density, chloroplast numbers, and levels of transcripts and proteins that are critical for photoperiodic function (72). Furthermore, changes in photosynthetic capacity have been tracked in natural environments, and photosynthetic rates are closely tied to daylength even when other environmental variables are present (6). In a particularly striking example, facultative crassulacean acid metabolism (CAM) plants, such as *Kalanchoe blossfeldiana*, switch from C_3_ photosynthesis to CAM photosynthesis when days shorten. This is likely due in part to the increase of phosphoenolpyruvate carboxylase protein levels and activity that are tied to SDs (11–13).

Not only is energy production controlled by photoperiod, but plants also exhibit exquisite control over the partitioning of photosynthetic assimilates. Many plants store starch, an osmotically inert storage polysaccharide, as an energy reserve in anticipation of unfavorable energy environments (125). More than 40 years ago, an inverse relationship between daylength and percent distribution of photoassimilates dedicated to starch was observed in soybean (19, 21). In LDs, a lower percent of the total photoassimilate is stored as starch, while in SDs this percentage increases. This likely serves as a transitory carbon storage system for the plant to produce mono- and disaccharide sugars in energy-limiting conditions, such as the long nights of SD conditions. Measurement of sugars bore out this hypothesis, showing that their presence is maintained at night when no new photoassimilate is being produced through photosynthesis. As mentioned previously, the term photoperiod was refined to photosynthetic period in this series of articles (19, 21) because this was a daylength measurement process that relied on metabolic rather than absolute daylength (19) (Figure 7).

*Arabidopsis* has emerged as a powerful model to study seasonal and daily control of photoassimilate distribution. The same transitory diurnal starch storage system exists in *Arabidopsis* as in soybean, but mutant analyses allow for investigating the molecular mechanisms involved in this system. For instance, the ability to accurately gauge night lengths requires a functional circadian clock, with clock mutants exhausting starch reserves before dawn or causing excess starch storage at night (36, 42, 44, 77, 131). Additionally, some of the first mutants ever identified in *Arabidopsis* were not able to produce starch, and these mutants are in a metabolic free run in which mono- and disaccharides are produced in the light but are quickly used up after dusk, causing the plant to enter a starvation state (16, 37, 105). This causes greater physiological defects when nights are longer, as in winter-like photoperiods, indicating the importance of the system for maintaining plant fitness across the year.

Recently, a series of carefully designed experiments showed that the percentage of photoassimilates dedicated to starch is dependent on daylength but independent of light intensity and the daily light integral, a so-called truly photoperiodic process that responds to the duration of the light rather than the photon flux intensity (89). This was subsequently shown to be under the control of the circadian clock when a *cca1 lhy elf3* triple mutant was unable to increase photoassimilate partitioning to starch in SDs, but maintained the ability to adjust starch degradation to accommodate the long night (3). This indicates that there are separate daylength measurement systems to alter starch synthesis and degradation rates in response to changing photoperiods. Interestingly, the MDLM system was shown to require appropriate starch synthesis and breakdown to accurately control expression of short and long day–specific genes (76, 145). It will be interesting to determine how photoperiodic regulation of starch occurs and how this is communicated to the MDLM system. After the discovery of the inverse relationship between daylength and starch accumulation rate in soybean, similar responses were found in spinach, sugar beet, corn, and pangola (20). However, in alfalfa, the percentage of photoassimilate dedicated to starch did not change under different photoperiods, suggesting that the connection between starch and photoperiod is not conserved among every plant species and can be discarded, similar to photoperiodic flowering in day-neutral plants.

The plant’s nutritional status is important for providing sufficient energy to produce viable seeds, and it has become clear that flowering is under control of the metabolic state of the plant. Early on, sucrose was observed to display a transient rise in the phloem near the shoot apex in *S. alba* in response to an inductive LD (7). Trehalose-6-phosphate (T6P), an important signaling sugar, correlates with sucrose levels across the daily light-dark cycle (35). In *Arabidopsis*, T6P peaks at the end of the day, and reduced T6P synthesis leads to delayed *FT* expression, whereas overproduction leads to early flowering in both SDs and LDs (143). Thus, T6P connects carbohydrate status to photoperiodic flowering and provides a tantalizing link between photosynthetic period and flowering. Profiling chromatin-accessible regions in single cells revealed that a shift to inductive LDs led to decompaction of chromatin proximal to the *TREHALOSE-6-PHOSPHATE SYNTHASE9* gene and concomitant upregulation (136). That being said, no role for T6P in flowering-time control has been demonstrated in other plants outside the Brassicaceae (34).

### 5.3. Growth Linked to Metabolism

Physiological studies of photoperiodic leaf growth have been performed in many species, and in general, rapid leaf expansion occurs in LDs, presumably because energy is replete, allowing the plant to dedicate resources to further increasing photosynthetic capacity. Opposite to this, hypocotyl growth in seedlings, which mostly consists of cell expansion, is greater in SDs, presumably to increase the height of the plant and give it a better chance to reach light. This process is controlled by the PIF photoperiod measurement system (102) (Figure 3; see also Section 4). Subsequently, vegetative growth allows for the expansion of photosynthetic tissues to acquire additional energy and is tightly linked to metabolism (42, 89). Historically, flowering and growth were both recognized to be under the control of photoperiod but able to be timed differentially throughout the year (138). For instance, both SD and LD plants can have an increased growth rate in LDs (142).

Plant growth can be influenced by many internal and external signals but at its most fundamental level requires cell division, cell expansion, and cell maintenance, and little is known about the molecular mechanisms that support photoperiodic leaf growth. In *Arabidopsis*, cell division and possibly cell expansion increase in LDs, resulting in rapid leaf growth prior to bolting (5, 24, 131). Interestingly, leaf initiation rate of *Arabidopsis* is not changed by photoperiod, but flowering is delayed in SDs, extending the phase of leaf initiation and resulting in a larger plant at bolting (24).

Growth rate in plants is often intimately linked to metabolic status, and the higher rate of photoassimilate partitioning to starch in SD-grown *Arabidopsis* was proposed to be caused by a restriction of growth rate (89). Additionally, the MDLM system was recently shown to promote LD expression of *MIPS1*, which is required to support leaf growth exclusively in LDs (145). Interestingly, the MDLM system also promotes SD expression of *PP2-A13*, which supports leaf growth in SDs, demonstrating that plants are taking account of their metabolic status in any season to optimize growth strategies with the proper time of year.

While not directly connected to any photoperiod measurement system, other mutants with photoperiod-specific growth defects have been reported. Autophagy mutants have SD-specific growth defects that are caused by salicylic acid overproduction (60). The mitochondrial FtsH4 protease protects against cellular death triggered by oxidative damage in SDs (63), and mutating a chloroplast NADPH-dependent thioredoxin reductase or a thiamine pyrophosphate riboswitch alters starch balance and causes SD-specific defects in growth (9, 73, 114).

### 5.4. CONSTANS-Dependent Growth Processes

Besides the photoperiodic control of vegetative growth in leaves and hypocotyl, many species have photoperiodic growth phenotypes linked to the protection of reproductive tissues and controlled by the CO/FT regulon.

#### 5.4.1. Short-day promotion of growth: tuberization.

Tubers are modified stems that act as important asexual reproductive structures, allowing a plant to survive underground during adverse environmental conditions. Potato tubers, for instance, allow a plant to overwinter underground, with the plant eventually reemerging from the meristematic potato eyes during favorable conditions (159). Night break experiments have demonstrated that tuberization in potatoes is controlled by photoperiod.

Similar to sexual reproduction through photoperiodic flowering, tuberization relies on a modified CO/FT regulon producing a mobile signal in the leaves (99). In LDs, *Solanum tuberosum CONSTANS-LIKE1* (*StCOL1*) peaks at dawn as a consequence of proteolytic degradation of the CDF1 repressor mediated by the FKF1/GI complex, and the StCOL1 protein accumulates due to stabilization by phytochromes. StCOL1 represses one of the *FT* paralogs, *StSP6A*, by transcriptionally activating the antagonist *StSP5G*. In SDs, CDF1 accumulates, inhibiting *StCOL1* expression and thus indirectly activating *StSP6A* and the transport of StSP6A to stolons to induce tuber formation (99).

According to this model, night breaks would inhibit tuber formation in SDs due to a stabilization of StCOL1 at night. More recently, it was shown that the repression of tuberization by night breaks cannot be solely explained by stabilization of StCOL1, implying additional factors beyond *StCOL1, StPG5,* and *StSP6A* in the photoperiodic control of tuberization (110).

#### 5.4.2. Short-day restriction of growth: growth cessation and dormancy in buds.

Perennial plants, especially those in boreal and temperate regions, have developed unique aboveground strategies to survive low temperatures in winter, including growth cessation and the formation of buds that have thick protective coatings that shield sensitive meristematic tissue from harsh winter conditions. Recently, new genetic and genomic technologies have propelled tree species such as hybrid aspen into the research community as models for studying overwintering strategies in perennial trees. These studies have demonstrated a clearly delineated set of steps that occur when inductive SDs are reached in autumn: The meristematic cells enter an energy-saving program, and progression of the cell cycle is reduced; plasmodesmatal transport is blocked, reducing the movement of growth-promoting compounds; buds begin to form; and then dormancy is established (140).

Akin to flowering and tuberization, short night breaks can slow or prevent entry into the dormancy developmental program that is controlled by a modified version of the canonical CO/FT regulon. LDs utilize the CO/FT regulon to induce flowering, similar to in *Arabidopsis*, but also to repress the dormancy-promoting gene *BRANCHED1* (*BRC1*) (84). This occurs via a transcriptional cascade beginning with activation of the *CO* ortholog. This in turn induces *FT2*, an *FT* ortholog, which activates the *LIKE-APETALA1* (*LAP1*) transcription factor gene. LAP1 then acts to repress *BRC1*, resulting in the promotion of growth and differentiation of meristems. In SDs, the *CO-FT-LAP1* regulon is reduced, resulting in the activation of *BRC1* and the establishment of the developmental transitions to dormancy.

Transcriptomics demonstrated that the establishment of winter bud formation requires the combined actions of abscisic acid and ethylene and results in decreased cell cycle progression and the reconfiguration of metabolic programming to meet the needs of a low-energy SD environment (115, 133). Similarly, in grapevine buds, each stage of dormancy and bud break across the year has distinct gene expression profiles that align with metabolic needs and stress responses (27).

#### 5.4.3. CONSTANS-dependent control of seed size.

Seed size is an agricultural trait that is particularly important for yield. Recently, it was shown that photoperiod controls seed size in a variety of species and that LD plants had larger seeds in LDs and SD plants had larger seeds in SDs (156). Accordingly, it was demonstrated that this photoperiod-specific effect on seed size required functional CO for photoperiod measurement. It was then shown that *CO* is expressed in the seed and acts to repress the gene *APETALA2*, which is known to repress seed size. This work is important because it further demonstrates that CO controls growth to protect reproductive and offspring tissues and that CO can function in seed tissues to control growth, independent of its role in flowering in the leaves.

### 5.5. Senescence

Senescence is a critical process for redistributing valuable resources in the plant. In annual species, senescence is used to pass nutrients to the seeds to ensure fitness in the offspring, while in perennials, resources are stored, often over winter, in roots or stems. It is important that senescence is tightly regulated. If senescence occurs too early, valuable photosynthetic tissue is lost, and if triggered too late, trees can experience marcescence or xylem embolism. Flowering often precedes senescence in SDs and LDs, indicating that senescence is under photoperiodic control, either directly or downstream of the CO/FT regulon (Figure 8). While very little is known about the molecular connections between photoperiod and senescence, in *Arabidopsis*, upregulation of the senescence-associated gene *SEN4* was higher in LDs than in SDs, which could act as a valuable marker to identify the molecular steps leading to photoperiodic control of senescence (64).

## 6. CLIMATE CHANGE AND PHOTIC BARRIERS TO LATITUDINAL MIGRATION

The climate catastrophe is rapidly disconnecting photoperiod from temperature and water availability, and recent work suggests that extinction of plant species will accompany changes in climate (31). To avoid extinction, it has been calculated that animal and plant species are tracking optimal thermal environments by moving poleward at a median rate of 16.9 km per decade, although species variability likely affects these rates dramatically (23, 98). Because photoperiod will not change as the climate warms, plants are attempting to align with thermal environments at the expense of remaining at optimal photoperiodic environments. While photoperiodic misalignment is less often considered in studies of latitudinal migration, a multitude of potential constraints have been proposed that could result in photic barriers, defined as obstacles in latitudinal movement due to photoperiod-dependent restrictions on fitness (53, 54, 137). Primarily, species with relatively strong photoperiodic responses, and less adaptive flexibility, are proposed to reach latitudinal photic barriers more quickly than highly adaptive species. This can cause misalignment with the thermal environment but also the biotic environment, constraining temporal or spatial alignment with pollinator species (124). Additionally, species with restricted range expansion are likely to face increased competition with invasive plant species or threat by pathogens that have expanded ranges caused by climate change (116, 144).

Many of the ancient relatives of our crops originated at the equator and are now grown at higher latitudes. Thus, relatively rapid latitudinal distribution was a key component of plant domestication, and we are now able to mine this genetic history to understand which challenges plants will face in the next 100 years of climate destruction. Genome-wide association studies of domesticated plants that have been crossed to their ancient counterparts tell us more about loci contributing to latitudinal movement. One cellular process that is often altered during domestication is the pacing of the circadian clock, which sets the timing for photoperiodic timekeeping (18, 75, 86, 158).

A clear example came from studies in day-neutral tomato, which offers the possibility to monitor the circadian clock and its response to photoperiod independently of photoperiodic flowering (95). Tomato originates from the Andean region. Early in the domestication process, the selection of loss-of-function alleles of tomato EMPFINDLICHER IN DUNKELROT1 (EID1), an F-box protein acting as a repressor of light signaling, led to a higher chlorophyll content in LDs and unintentionally delayed the circadian clock phase so that physiological processes occurred later during the light-dark cycle. EID1 has a large effect on the response of the transcriptome to short photoperiods, likely due to the global phase shift EID1 exerts on circadian rhythms (151).

Variations in photoperiodic flowering are a key factor when crops are introduced in agricultural areas far from their origin upon domestication. *H. vulgare*, originating from the Fertile Crescent in the Middle East, carries an active allele of *Photoperiod-H1* (*Ppd-H1*), a pseudo-response regulator and activator of flowering, and flowers early in spring in LDs before the onset of summer droughts (139). By contrast, barley cultivars grown at higher latitudes, including northern Europe, display loss-of-function *ppd-H1* alleles and show a reduced response to LDs, extending the growth period and the yield potential.

Rice originates from subtropical regions, and its adaptation for growth in higher latitudes selected for reduced photoperiod sensitivity. Loss-of-function mutations in LD-specific floral repressors, including *Ghd7, DTH8,* and *Hd1*, correlate with northward expansion (147, 152, 154). Similarly, the natural variation of floral activators, including *DTH2* and *Ehd4*, contributes to adaptation to high latitudes (38, 150). In the planting areas of origin, reduced photoperiod sensitivity allows more than one round of planting. Now research has moved its focus to accelerating latitudinal movement to preempt the effects of climate change. To speed up the traditional breeding program for rice varieties optimally suited for the corresponding latitude and to readjust the heading date, Qui et al. (112) have developed a daylength-based environmental adaptation simulator, inferring latitudinal adaptation based on florigen expression. In a large-scale experiment, rice plants were exposed to simulated changes in daylength in growth chambers and *Hd3a* and *RFT1* levels were monitored. The dynamics of *Hd3a* and *RFT1* expression in the natural daylength at a particular latitude could be inferred, and thus heading behavior can be predicted. Importantly, additional incorporation of fluctuating temperatures may further increase the predictive power.

We suggest that basic understanding of photoperiod measurement systems will be critical for reactive and proactive strategies to combat the climate disaster. This should be combined with gaining more knowledge of the genetic history associated with domestication across latitudes to design engineering strategies for breeding crops for the future. In particular, manipulating components of the circadian clock may provide a means to delay the phases of clock-controlled processes, including variation of the responsiveness to biotic and abiotic threats throughout the day, to match longer days when a species moves northward.

## 7. CONCLUSIONS AND OUTLOOK

Plant species continue to serve as the vanguard for understanding photoperiodism. There is no other system with a more complete molecular model of a timekeeping mechanism than the CO/FT regulon. This puts the field in a unique place in which engineering strategies become feasible. There is one area that needs to be investigated more deeply—the interactions between photoperiodic flowering and other environmental cues. For example, measuring flowering time in *Arabidopsis* under ecologically realistic conditions revealed that major genes underpinning flowering time in controlled growth chamber experiments were not associated with flowering time in the field. Instead, genes involved in the regulation of the circadian clock correlated with flowering time (10). More recently, in addition to the *FT* peak at the end of the day in spring, field studies unearthed a second *FT* peak in the morning, which is not found under laboratory conditions (127). Adjusting the red/far-red ratio and the daily temperature in growth chambers to the situation found in the field is sufficient to reproduce natural flowering time and *FT* expression, enforcing the importance of seasonal changes in temperature as a critical factor.

It is also clear that the genomics revolution is a powerful component of the photoperiod research field. We recommend considering among best practices in experimental design the acquisition of high temporal resolution over at least one day, preferably with multiple replicates for each day. This allows for temporally unbiased assessment of the genes that have phase or amplitude changes in the different photoperiods. This will allow us to construct similar types of expression cascades, as established for the CO/FT regulon (Figure 5), with other noncanonical photoperiod-measuring systems.

The discovery of noncanonical photoperiod-measuring systems dispels the idea that the CO/FT regulon can account for the totality of photoperiodic changes in plants (Figure 3). The construction of tools and generation of transcriptomic and epigenomic data will facilitate the discovery of even more photoperiod-measuring systems. Until then, additional effort must be placed in understanding the molecular constituents of the MDLM and PIF systems as well.

It has also become clear that plants are distinguishing between absolute and metabolic daylengths (Figure 7). The CO/FT and PIF systems are measuring absolute daylength, while MDLM and the starch accumulation systems are measuring the photosynthetic period. This raises the interesting possibility that any type or quality of light that has seasonal variation may have associated photoperiod measurement systems in plants. Thus, it is helpful to categorize cellular processes by the type of photoperiod that is being measured and what timekeeping system is measuring the photoperiod to avoid confusion.

While it is clear that multiple steps in development are controlled by photoperiod, we have sparse knowledge of how this may work. Developing photoperiod-specific phenotypes to assay for various developmental steps will be a good first step for pursuing the photoperiod-measuring system that controls each process.

Finally, the efforts within the field have opened the door for us to engineer climate change–resistant plants, but one may want to ask if the plants can help us in return. Currently, synthetic carbon capture systems have not reached the level and efficiency of plant carbon capture. Because photoperiod has such a strong effect on carbon capture, storage, and usage in plants, we may possibly be able to manipulate photoperiod measurement systems in plants to engineer increased carbon capture in crops.

## SUMMARY POINTS

1. Classical models have implicated an endogenous rhythm of light sensitivity as the basis of photoperiodic flowering-time control, according to the coincidence of ambient light with either a light- or dark-sensitive phase of this rhythm.
2. Molecular genetic studies in *Arabidopsis* were the first to show that CONSTANS (CO) represents the key factor mediating photoperiodic flowering-time control by activating the florigen *FLOWERING LOCUS T* (*FT*). The CO/FT regulon provides a framework with features of both the external and internal coincidence mechanism. The CO/FT regulon also functions in photoperiodic timekeeping in short-day plants where the activity of CO-like proteins is regulated oppositely.
3. Photoperiodic responses in natural settings with a plethora of environmental factors that vary throughout the seasons differ from photoperiodic responses observed in growth chambers with controlled conditions. In particular, temperature fluctuations appear to have a decisive effect.
4. Photoperiod transcriptome studies reveal gene expression cascades associated with photoperiodic flowering. Additionally, they help identify new molecular mechanisms, show there are likely more photoperiod timekeepers in plants, and reveal the many cellular and physiological processes being regulated by photoperiod.
5. Not all photoperiodic responses can be explained by the CO/FT regulon. The phytochrome-interacting factor (PIF) and metabolic daylength measurement (MDLM) photoperiod-measuring systems track daylength to control growth.
6. Plants can measure absolute photoperiod to control flowering and photosynthetic period to control starch accumulation, the MDLM system, and growth.
7. Many steps in plant development are controlled by photoperiod, but little is known about the regulons that control these processes.
8. Studying genetic modifications associated with domestication can help guide our actions to ameliorate the effects of climate change.

## Abbreviations

Photoperiod: duration of the light phase within a day
Photoperiodism: use of daylength sensing to adjust molecular, biochemical, and physiological processes to the course of the seasons
Photosynthetic period: photoperiod that begins when light intensity rises above the compensation point and ends when light intensity drops below the compensation point
Phytochrome: a photoreceptor occurring in two states: the inactive Pr form and the active Pfr form
Short-day plant (SD plant): a plant that flowers when daylength remains below the critical daylength
CONSTANS (CO): a transcription factor expressed in phloem companion cells of leaves that is controlled by the circadian clock and mediates activation of florigen genes in inductive photoperiods
FLOWERING LOCUS T (FT): a mobile protein that in inducing photoperiods is transported from leaves to the shoot apical meristem via the phloem to trigger flowering
Shoot apical meristem (SAM): tissue at the shoot tip that contains undifferentiated cells and, upon the arrival of a floral stimulus, initiates flower formation
Florigen: a classical term referring to a mobile substance produced in leaves under inductive conditions that transfers the photoperiodic information to the shoot apical meristem to evoke floral transition
Long-day plant (LD plant): plant that flowers when daylength exceeds the critical daylength
Critical daylength: duration of daily light period required to induce flowering of long-day plants or to inhibit flowering of short-day plants
Night break: light pulse applied during the long, dark period of a short day that promotes flowering of long-day plants and prevents flowering in short-day plants
Metabolic daylength measurement (MDLM) system: photoperiod-measuring system based on the photoperiod-controlled regulation of starch degradation
Absolute photoperiod: photoperiod that begins with the first photons of light at dawn and ends with the last photons of light at dusk
Compensation point: the light intensity at which the rate of photosynthesis is equal to the rate of respiration; at this light intensity, the net carbon fixation rate is equal to zero
PHYTOCHROME-INTERACTING FACTORS (PIFs): transcription factors that interact with active phytochrome
Photoassimilate partitioning: allocation of products of photosynthesis for the synthesis of sucrose or starch
Photic barrier: a barrier to latitudinal or altitudinal movement caused by unfavorable light conditions
Domestication: cultivation of wild species in new environments to meet human needs, entailing genetic changes and generating new varieties better adapted to zones suitable for agriculture
